# Between Scylla and Charybdis

**DOI:** 10.1007/s12471-019-1289-3

**Published:** 2019-05-21

**Authors:** H. J. te Kolste, G. J. Kimman, T. Germans, S. A. J. Timmer

**Affiliations:** 1 Location VU Medical Center, Amsterdam University Medical Center, Amsterdam, The Netherlands; 2 North-West Hospital Group, Alkmaar, The Netherlands

A 69-year-old patient presented at the emergency department after experiencing near-syncope. He was known at the outpatient clinic with a Wolff-Parkinson-White (WPW) pattern seen on electrocardiogram (ECG) and was previously asymptomatic. The ECG at rest showed sinus rhythm with right bundle branch block (RBBB) and left anterior fascicular block (LAFB), alternating with a pre-excited QRS-complex morphology consistent with a left lateral and/or anterolateral insertion of the accessory pathway (Fig. 1). Additional investigations (laboratory studies, echocardiography) were unremarkable. During 48-hour telemetric observation no impulse or conduction abnormalities (other than intermittent pre-excitation) and no tachyarrhythmias were observed. Exercise testing resulted in aggravation of ventricular pre-excitation at increasing heart rate (>100 bpm). Subsequently, the patient was started on flecainide to increase refractoriness of the accessory pathway and was scheduled for electrophysiological studies. Six weeks later, the patient suffered from syncope resulting in a fracture of the left humerus. What does the ECG on admission show (Fig. 2)?

**Fig. 1:**
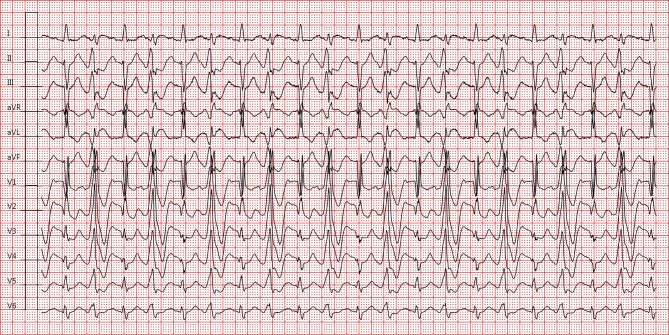
Electrocardiogram at rest after near-syncope

**Fig. 2:**
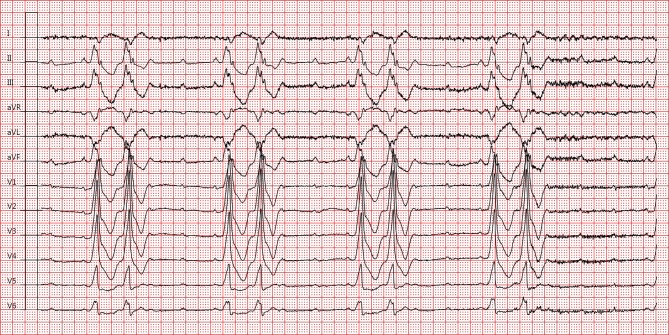
Electrocardiogram on admission after syncope

## Answer

You will find the answer elsewhere in this issue.

